# Causal modulation of right hemisphere fronto-parietal phase synchrony with Transcranial Magnetic Stimulation during a conscious visual detection task

**DOI:** 10.1038/s41598-020-79812-y

**Published:** 2021-02-15

**Authors:** Chloé Stengel, Marine Vernet, Julià L. Amengual, Antoni Valero-Cabré

**Affiliations:** 1 Cerebral Dynamics, Plasticity and Rehabilitation Group, FRONTLAB team, Institut du Cerveau et la Moelle Épinière (ICM), Pitié-Salpêtrière Hospital, CNRS UMR 7225, INSERM U 1127 & Sorbonne Université, 47 boulevard de l’Hôpital, Paris 75013, France; 2 IMPACT Team, Lyon Neuroscience Research Center (CRNL), CNRS UMR 5292, INSERM UMRS 1028, University Claude Bernard Lyon 1, Lyon, France; 3 Institut Des Sciences Cognitives Marc Jeannerod, CNRS UMR 5229, Université Claude Bernard Lyon 1, Lyon, France; 4 Laboratory for Cerebral Dynamics Plasticity and Rehabilitation, School of Medicine, Boston University, Boston, MA USA; 5 Cognitive Neuroscience and Information Technology Research Program, Open University of Catalonia (UOC), Barcelona, Spain

**Keywords:** Attention, Perception

## Abstract

Correlational evidence in non-human primates has reported increases of fronto-parietal high-beta (22–30 Hz) synchrony during the top-down allocation of visuo-spatial attention. But may inter-regional synchronization at this specific frequency band provide a causal mechanism by which top-down attentional processes facilitate conscious visual perception? To address this question, we analyzed electroencephalographic (EEG) signals from a group of healthy participants who performed a conscious visual detection task while we delivered brief (4 pulses) *rhythmic* (30 Hz) or *random* bursts of Transcranial Magnetic Stimulation (TMS) to the right Frontal Eye Field (FEF) prior to the onset of a lateralized target. We report increases of inter-regional synchronization in the high-beta band (25–35 Hz) between the electrode closest to the stimulated region (the right FEF) and right parietal EEG leads, and increases of local inter-trial coherence within the same frequency band over bilateral parietal EEG contacts, both driven by *rhythmic* but not *random* TMS patterns. Such increases were accompained by improvements of conscious visual sensitivity for left visual targets in the *rhythmic* but not the *random* TMS condition. These outcomes suggest that high-beta inter-regional synchrony can be modulated non-invasively and that high-beta oscillatory activity across the right dorsal fronto-parietal network may contribute to the facilitation of conscious visual perception. Our work supports future applications of non-invasive brain stimulation to restore impaired visually-guided behaviors by operating on top-down attentional modulatory mechanisms.

## Introduction

High-level cognitive functions, such as spatial attention orienting or access to perceptual consciousness, cannot solely rely on the activity of single cortical regions but require the integration of processes occurring across widely distributed cortical nodes organized in complex brain networks^1,2^. Accordingly, understanding how distant cortical regions communicate as part of a single distributed network during the performance of a cognitive task has become a crucial mission for the systems neuroscience field.

Early theories of inter-regional communication in the human brain focused on the study of white matter connectivity, considered to be the backbone of long-distance communication^3,4^. However, neuronal activity and patterns of functional connectivity show episodic dynamic fluctuations operating in the order of milliseconds^5,6^. Consequently, these cannot be solely characterized by the architecture of inter-areal structural pathways (a.k.a., the connectome), which is lacking the flexibility for a dynamic and selective communication between subsets of brain systems organized as nodes within highly interconnected networks^7^.

For the last two decades, new mechanistic models have proposed that local and inter-regional communication between neuronal populations is subtended by the synchronization of their oscillatory activity^1,7–10^. Such models have claimed that when two natural cortical oscillators synchronize in frequency and/or in phase, the spikes generated by a first group of neurons will reach well-synchronized neurons within a target population at their peak of excitability, ensuring a higher gain in information transfer and, consequently, more efficient communication. This so called model of *communication-through-coherence*^7,8^ has been hypothesized to be particularly important in mediating top-down modulations (e.g. by attentional or perceptual processes) of inputs signals entering primary sensory areas^10^.

Experimental data in support of long-distance synchronization during visual perception and the orienting of attention have been collected both in animal models^11–13^ and humans^14–16^. The former evidence suggests that, in such processes, fronto-parietal regions synchronize at a beta or gamma frequency band (ranging from 15 to 60 Hz) during episodes of attentional orienting or perception. However, these reports associated synchronization with specific behaviors solely on the basis of their co-occurrence in time, an approach which has proven unable to distinguish causal contributions of oscillatory activity from potential epiphenomena.

 Transcranial Magnetic Stimulation (TMS), a non-invasive technology able to stimulate circumscribed cortical regions, in combination with Electroencephalography (EEG), allows  to probe the causal implication of oscillations and synchronization patterns between cortical locations in behavioral effects likely encoded through such mechanisms. Indeed, TMS has demonstrated the ability to non-invasively manipulate attentional and perceptual behaviors^17–21^ as well as local and network activity correlates^22–25^ by inducing, interfering or modulating ongoing neural signals from circumscribed cortical sites of the human brain.

More recently, it has also been shown that the delivery of brief bursts of TMS pulses (usually 4–5 pulses) regularly spaced in time (a stimulation modality  known as rhythmic TMS), structuring a regular alpha rhythm around ~ 10 Hz (i.e. ~ 1 TMS pulse every 100 ms), progressively phase-locked natural alpha oscillators over the posterior parietal cortex in human brains not enganged in any specific task^26^. Ensuing studies have reported evidence supporting the ability of rhythmic TMS, applied in a wide range of frequency bands, to modulate performance in different cognitive processes^20,27–29^. Finally, a recently published report by our group provided evidence that 30 Hz rhythmic TMS delivered over the right Frontal Eye Field (FEF), a key region of dorsal attention orienting networks, was able to locally entrain high beta oscillations in the frontal region below the stimulation coil. Most importantly, it suggested that such local entrainment was causally linked to improvements of conscious visual detection for left-lateralized near-threshold targets^30^.

Through the above mentioned studies, rhythmic TMS has built a solid credibility as a tool enabling the causal exploration  of the oscillatory mechanisms underlying the top-down modulation of conscious visual perception in humans by identifying performance shifts tied to the entrainment of local rhythmic activity at specific frequency bands and cortical sites. However, the mechanisms subtending the modulation of the inter-regional synchrony between a TMS-stimulated region (in the current study the right FEF) and other nodes of the dorsal attentional orienting network, and the involvement of such activity patterns in the modulation of conscious visual perception during the delivery of rhythmic TMS pulses remain rather unknown.

In this context, building on evidence showing that TMS-evoked oscillations can spread through connections to distant regions of the same network^31^, we further analyzed data from a recent  TMS-EEG study published  by our lab^30^, aiming to extend prior results supporting a modulatory role on conscious visual perception  for local episodic entrainment of high-beta activity in the right FEF. We hypothesized that the brief entrainment of 30 Hz oscillations by rhythmic TMS patterns on this cortical region would induce transient inter-regional phase synchronization at a high beta frequency, likely within a dorsal fronto-parietal system linking this frontal site with posterior parietal areas^32–34^. We anticipated that such hypothesis would be substantiated in EEG recordings by higher 30 Hz phase-locking values between the EEG contact closest to the stimulated frontal site on the right FEF and posterior parietal EEG contacts during *rhythmic* compared to *random* TMS patterns. We also hypothesized that, through increased fronto-parietal phase synchronization, rhythmic stimulation over the right FEF would entrain high-beta oscillations in ipsilateral posterior parietal cortical locations. Substantiating these effects, we predicted increases of local high beta power and inter trial phase coherence (ITC) over right posterior parietal electrodes during *rhythmic* compared to *random* TMS patterns.

Crucially, our study was based on a carefully designed TMS control condition (based on the application of *random* TMS patterns) delivering the same number of TMS pulses, thus the same amount of stimulation as our *rhythmic* 30 Hz TMS patterns of interest, but lacking a regular frequency-specific spacing between  pulses. As shown in prior publications, this strategy enabled us to isolate the effect of the rhythmicity of the stimulation on the entrainment of local oscillations as well as﻿ on inter-regional synchronization^18,30,34^.

## Material and methods

### Participants

We performed new analyses on an existing TMS-EEG dataset from a recent publication that demonstrated local high-beta entrainment during the stimulation of the right FEF and  associated improvements of conscious visual detection performance (see ref 30 for details). A group of 14 right-handed healthy participants (9 women) aged between 20 and 34 years old (24 ± 4, mean ± SD) took part in the original experiment. Participants had normal or corrected-to-normal vision. They all participated voluntarily after providing written informed consent and were naïve as to the purpose of the experiment. All the experimental procedures were performed according to the Declaration of Helsinki. A research protocol including all the interventions of this study was sponsored by the INSERM and approved by an Institutional Review Board known in France as *Comité de Protection de Personnes* (CPP Ile-de-France IV).

### Conscious visual detection paradigm

An in-house MATLAB (Mathworks, version R2012b) script using the Psychtoolbox extensions^35^ was used to control the presentation of visual stimuli synchronized with the delivery of the TMS pulses. During the task, participants were seated with their heads resting on a chin-rest set so that their eye’s canthi remained 57 cm away from the center of the screen.

**Figure 1:**
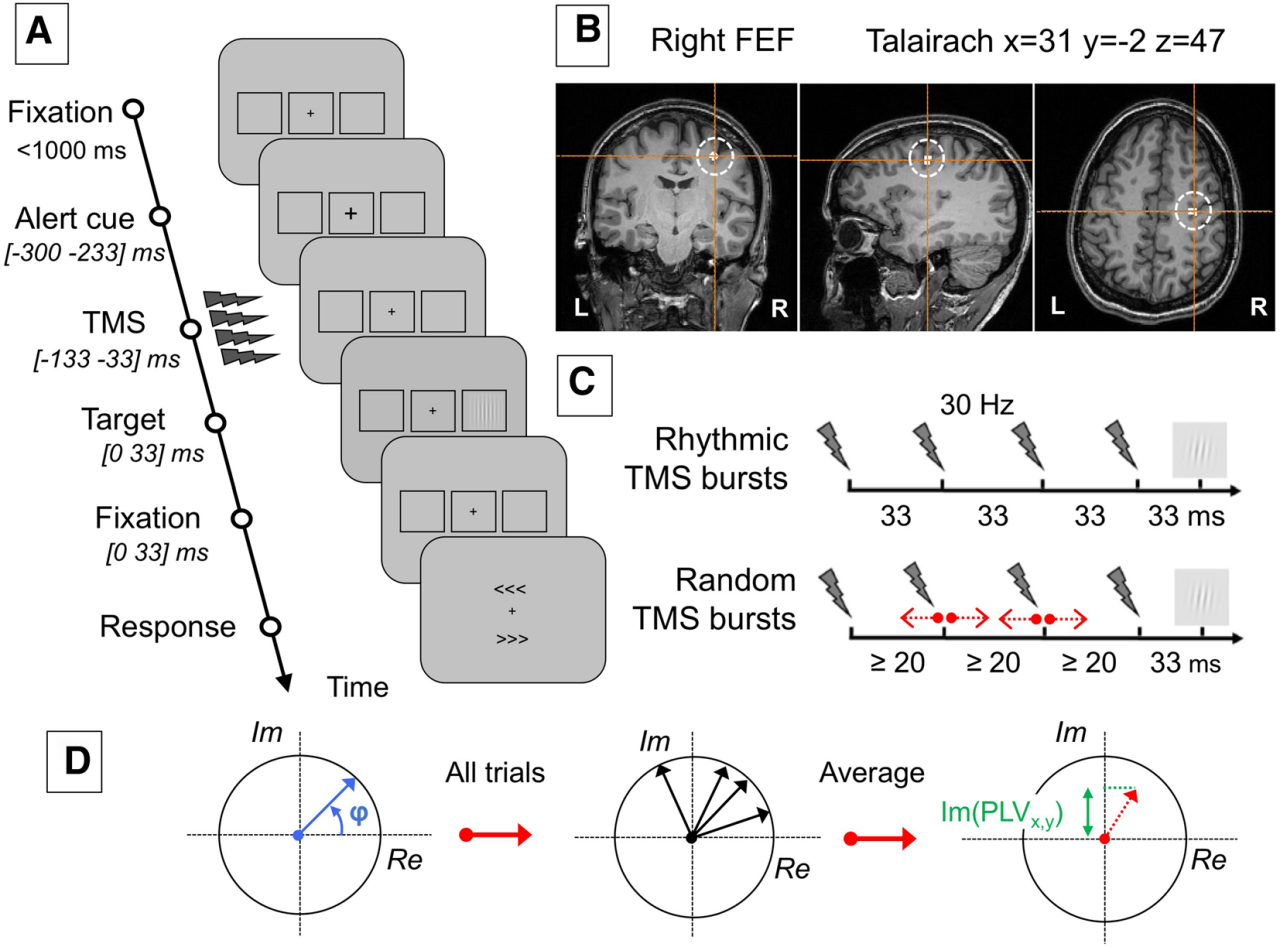
Experimental design, targeted cortical regions, TMS stimulation patterns and EEG phase synchrony measures. (**A**) Computer-based visual detection task performed by participants. After a period of fixation, a central cross became slightly larger to alert participants of an upcoming event; then active and sham *rhythmic* or *random* TMS patterns were delivered to the right FEF region prior to the presentation of a target that could appear at the center of a right or left placeholder for a brief period of time. Participants were requested to indicate whether they did perceive a target or not and, if they saw it, where it appeared (right/left). Notice that in 20% of the trials, no target was presented in any of the placeholders. (**B**) Coronal, mid-sagittal and axial MRI sections from the frameless stereotaxic neuronavigation system showing the localization of the targeted cortical right FEF (Talairach coordinates x = 31, y = − 2, z = 47) in a T1-3D MRI of a representative participant. (**C**) Schematic representation of the active and sham 4 pulse TMS patterns employed: 30 Hz *rhythmic* stimulation to entrain oscillatory activity at the input frequency (~33 ms interpulse interval) in the right FEF, and the *random* stimulation (jittered interpulse intervals) used as a control to isolate the effect of stimulation frequency. (**D**) Schematic representation of phase locking value calculations to estimate changes in interregional synchronization. The phase of signals between two sets of electrodes was extracted from the Fourier spectra. For all trials, complex vectors were reconstructed with a phase (φ) equal to the phase difference between the two signals. These complex vectors were averaged over trials. TMS volume conduction was ruled out by calculating the imaginary phase locking value (imaginary PLV or imPLV), which consisted in the projection on the imaginary axis of the complex vector averaged over all trials.

Each trial started with a gray resting screen lasting for 2.5 secs, followed by a fixation screen that displayed a central fixation cross (size 0.5 × 0.5°) and right and left rectangular placeholders (6.0 × 5.5°) drawn 8.5° away from the center (Fig. 1A). These placeholders indicated the potential right or left lateralized locations of the target during the trial. The duration of the fixation screen was jittered between 1.0 and 1.5 secs to avoid predictability with regards to upcoming events. A brief-lasting (66 ms) size increase (0.7 × 0.7°) of the central fixation cross alerted participants of the presentation of an upcoming target. After an inter-stimulus interval of 233 ms, in 80% of the trials a target appeared in the middle of the left or the right placeholder with equal probability. The remaining 20% of the trials were ‘catch’ trials in which no target was shown in any of the placeholders. The target consisted of a low-contrast Gabor stimulus with vertical lines (0.5°/cycle sinusoidal spatial frequency, 0.6° exponential standard deviation), appearing for 33 ms. Stimulus contrast was individually adjusted for each participant during a calibration block carried out prior to the onset of the experimental session. At all times, contrast level was never set below 0.005 Michelson contrast units. Similar tasks had been employed in prior TMS publications by our research group^18,30,34,36^.

Participants were asked to perform a conscious detection task, in which they had to report if they saw the target and, if ‘yes’, to indicate on which side it appeared (‘right’ or ‘left’). The response window consisted in two arrow-like signs (“ >  >  > ” and “ <  <  < ”) presented simultaneously below and above the fixation cross signaling the location of the right and left rectangular placeholders. Participants were requested to indicate which arrow pointed to the location of the placeholder where they had seen the Gabor target. The location of each arrow was randomized across trials to prevent the preparation of a motor response prior to the appearance of the response window and to make sure that visual processing activities in the FEF, located close to motor areas, were temporally segreated from motor decisions and finger responses. Participants provided a response using three keyboard keys: an upper key to indicate the upper arrow (corresponding to the ‘d’ letter key), a lower key to indicate the lower arrow (corresponding to the ‘c’ letter key) and the space bar to report that no target had been consciously perceived.

Participants performed 6 blocks: 1 calibration block, 1 training block and 4 experimental blocks (2 blocks for each TMS pattern: *random* and* rhythmic*, see details on TMS patterns below). The order of the experimental blocks was counterbalanced across participants. Each block was divided into sub-blocks of 20 trials. The length of the calibration and training blocks was variable, as the termination of these two blocks depended on individual performance. Experimental blocks consisted of 7 sub-blocks and lasted approximately 20 min each.

During the calibration block, target contrast was adjusted to reach a performance of 50% correct detections using a staircase procedure^37^. Initially, the Gabor contrast was set very high (Michelson contrast of 1). Then, on each trial, target contrast increased or decreased according to the response of the participant. The initial step in contrast was equal to the initial contrast level (note that, regardless, contrast was always kept higher than 0.005 Michelson contrast). On each reversal, contrast steps were divided by two. When, in five consecutive trials, contrast varied by less than 0.01 Michelson contrast, we considered that the 50% detection threshold had been reached. The threshold was measured a second time using the same procedure. The two thresholds were then compared. If they differed by less than 0.01 Michelson contrast, the calibration block was terminated and the final contrast level used during the following blocks was the average between the two thresholds. If they varied by more than 0.01 Michelson contrast, the threshold was measured again. During the calibration block, only sham TMS patterns were delivered on participants’ right frontal cortex. At the end of each sub-block, participants were invited to take a short break.

Before starting the experimental blocks, participants underwent a training block during which trials with active TMS (see below for further detail on TMS procedure) were introduced. For all conditions (no target present, target on the right placeholder, target on the left placeholder) half the trials delivered active TMS patterns whereas the other half delivered sham TMS patterns. The order of presentation of active and sham TMS was randomized for each sub-block of 20 trials. Participants’ performance during the training block was verified to ensure that it remained stable even with the intermixed active TMS trials. At the end of each sub-block in this training period, participants were alerted if their false alarm rate was higher than 50% and received feedback on the percentage of incorrectly reported target position and incorrect fixations. Between sub-blocks, the experimenter could also manually adjust the target contrast. Once participants reached a stable detection performance, the experimenter decided to end the training blocks and start the experimental blocks. These were identical to training blocks (with the same feedback for the participants) except that target contrast was kept constant  throughout all sub-blocks and participants were allowed to take a short break only every two sub-blocks.

### Recording of eye movements

During all blocks, the position of both eyes was monitored on each participant with a remote eye tracking system (Eyelink 1000, SR Research, at sampling rate of 1000 Hz). If the location of the participant’s eyes was recorded more than 2° away from the center of the fixation cross at any time between the appearance of the alerting cue (central cross increasing in size) and Gabor target offset, the trial was considered as non-fixated. The trial was re-randomized amongst the remaining trials in the sub-block and repeated. Non-correctly fixated trials were excluded from any subsequent data analysis.

### TMS procedure

TMS was triggered in synchronization with the presentation of the visual stimuli via a high temporal resolution multichannel synchronization device (Master 8, A.M.P.I.) connected to two biphasic repetitive stimulators (SuperRapid, Magstim) each attached to a standard 70 mm diameter figure-of-eight TMS coil. The coil in charge of delivering active TMS patterns was held tangentially to the skull above the location of the right FEF, with its handle oriented ~ parallel to the central sulcus at a ~ 45° angle in a rostral to caudal and lateral to medial direction. The other TMS coil, which delivered sham TMS stimulation was placed close to the stimulation site but positioned perpendicular to the skull, hence directing the magnetic field away from the brain. The sham coil produced the same clicking noise characterizing the delivery of an active TMS pulse but did not project any active hence effective stimulation to the targeted right frontal cortex.

During the whole experiment, the position of the active TMS coil was tracked using a MRI-based frameless stereotaxic neuronavigation system (Brainsight, Rogue Research). A T1-weighted MRI scan (3 T Siemens MPRAGE, flip angle = 9, TR = 2300 ms, TE = 4.18 ms, slice thickness = 1 mm) was acquired for each participant and the right FEF was localized on each individual scan as a spherical region of interest of 0.5 cm radius centered on the Talairach coordinates x = 31, y = − 2, z = 47^38^ (Fig. 1B). Using this neuronavigation system the active TMS coil was kept within a ± 3 mm radius from the center of the targeted site during the whole experimental session.

As done previously^18,30,34^, the two types of TMS patterns employed in this experiment consisted in a burst of four TMS pulses, lasting 100 ms and ending 33 ms before the onset of the visual target. Two types of patterns were tested: a *rhythmic* pattern for which the pulses were delivered regularly at a frequency of 30 Hz (i.e., ~33 ms of inter-pulse interval within the burst) and a *random* pattern engineered not to deliver any specific or pure single frequency.  In the * random* patterns, the onset timings of the 1st and the 4th pulse within the burst and total burst duration (100 ms) were kept identical to those of rhythmic patterns. However, the 2nd and 3rd pulses were randomly jittered before and after their exact onset timings in *rhythmic* 30 Hz patterns (Fig. 1C). Some additional constraints applied to pulse timing randomization for ﻿*random* patterns﻿. First, since the time needed by the TMS capacitors to fully re-charge before delivering the next pulse was limited, two pulses could not be delivered less than 20 ms apart. Second, the onset time of the two middle pulses (the 2nd and the 3rd) had to be shifted at least 3 ms away from the timings of these same pulses in the *rhythmic* pattern, to ensure that *random* patterns would never deliver a perfectly regular 30 Hz frequency.

TMS stimulation intensity was set at a fixed level of 55% of the maximal simulator output for all participants. This level was slightly higher than the intensity proven efficient in prior studies by our team^18,33,36^ to take into account the increased distance between the TMS coil and the cortex due to the presence of the EEG electrodes and the cap. To allow across-study comparisons, at the end of the experiment, the individual resting motor threshold (RMT) in the right hemisphere was determined for each participant as the TMS intensity that yielded a motor activation of the *abductor pollicis brevis* muscle (thumb motion) in about 50% of the attempts^39^. On average, the RMT was at 72 ± 9% (mean ± SD) of maximum stimulator output. The fixed stimulation intensity (55% of maximal machine output) used for TMS corresponded to 78 ± 12% (mean ± SD) of the participants’ individual motor thresholds.

### EEG recordings

EEG signals were continuously recorded during all experimental blocks with a TMS-compatible system (BrainAmp DC and BrainVision Recording Software, BrainProducts GmbH). We recorded signals from 60 electrodes spread evenly across the scalp and positioned according to the international 10–20 system, plus a reference on the tip of the nose, a ground on the left ear lobe and 4 additional EOG electrodes positioned above and below the right eye and on each temple. Skin/electrode impedances were maintained below 5 kOhm. The signal was digitized at a sampling rate of 5000 Hz.

#### EEG epoching and artifact removal procedure

All EEG data analyses were performed with the FieldTrip toolbox^40^ running on MATLAB R2017b. The EEG and EOG data were epoched across a [− 2, 2] seconds window centered on visual target onset (t=0). Trials in which the participant did not correctly fixate the central cross during the time interval between the alerting cue onset and the target offset were automatically excluded during the task performance by monitoring gaze position using an eye tracking system (see above for further details). Prior to any data analysis, all trials contaminated by blinks in the [− 500 500] ms epoch around visual target onset were removed by visual inspection. After these procedures, an average of 126 ± 13 (mean ± SD) trials remained for each experimental block.

To remove the artifact created by the discharge of each TMS pulse, EEG data in a window of [− 4, + 12] ms centered on pulse onset timing  were removed. A second order Butterworth filter (1 to 50 Hz), with forward-backwards filtering, was applied on the remaining data before the gap of removed artifacted EEG data was filled with a piecewise cubic spline interpolation. To reduce the burden of computation time, EEG data were down-sampled to 500 Hz prior to the application of an Independent Component Analysis (ICA). To make sure that the ICA did not introduce any artificial difference between TMS conditions, trials for all 4 experimental blocks (whether they were active* active* or *sham* TMS trials, and regardless of the tested TMS pattern, *rhythmic* or *random*) were gathered together and the ICA was computed on this single dataset. This procedure enabled the removal of residual artifacts (including eye movements, electrode malfunctions, 50 Hz power line artifacts and TMS artifacts lasting longer than 12 ms post pulse onset). Components were identified as artifacts based on the guidelines of Rogasch et al.^41^. On average 9 ± 2 (mean ± SD) components were rejected. After this procedure, data was separated into four conditions: Active *rhythmic* TMS, Sham *rhythmic* TMS, Active *random* TMS and Sham *random* TMS. We here made sure our data cleaning procedure completely removed TMS artifacts so that remaining electromagnetic artifacts could not account for statistically significant effects observed between differet types of TMS bursts reported in the current study (see Supplementary Materials, Figs. S1 and S2 for full details).

#### TMS-EEG data analyses

Epoched EEG signals were transformed into the time–frequency domain using a 3-cycle Morlet wavelet transform on the time window [− 500 + 500] ms around visual target onset (t=0) and for frequencies between 6 and 50 Hz. Three measures associated to the notion of oscillation synchronization were calculated: power, inter-trial coherence (ITC) and imaginary phase-locking value (imaginary PLV or imPLV). Power was calculated as the squared value of the modulus of the Morlet coefficients (per each time frame and frequency bin) relative to a baseline window [− 500 − 300] ms before visual target onset. This outcome measure shows, in decibels (dB) units, the increase (positive values) or decrease (negative values) of power relative to this baseline period.

Inter-trial coherence (ITC) estimates phase consistency across trials in a given location (an electrode or a group of electrodes). To measure synchronization between distant regions, we calculated the phase-locking value (PLV)^42^. This measure reflects the stability of the phase difference between two signals in a specific frequency band. The phase difference between two signals is extracted at all points in time from their Fourier spectra and is then averaged across all trials. The PLV is defined as the module of the resulting averaged complex vector across trials (Fig. 1D). The following formula was used for its computation:

$$PLV_{x,y} \left( t \right) = \left\| {\frac{1}{{n_{trials} }} \cdot \mathop \sum \limits_{trials} e^{i \cdot \varphi \left( t \right)} } \right\|$$

where $\varphi \left( t \right)$ represents the phase difference between the signal recorded by the electrodes *x* and *y* at time *t* and *n*_*trials*_ is the total number of trials in the condition.

The PLV, as the module of a unit vector, is always comprised between 0 (random phase difference between the two signals) and 1 (constant phase difference between two signals)^43^. To avoid controversy on the fact that the PLV might be very sensitive to volume conduction, we considered as a synchrony value the projection on the imaginary axis of the complex vector of the PLV, hence the so called imaginary PLV^44,45^.

$${\text{Im}} \left( {PLV_{x,y} \left( t \right)} \right) = {\text{Im}} \left( {\frac{1}{{n_{trials} }} \cdot \mathop \sum \limits_{trials} e^{i \cdot \varphi \left( t \right)} } \right)$$

Volume conduction is a phenomenon that affects scalp EEG data when signals from a single cortical source are recorded simultaneously by several sensors on the scalp, hence leading to higher values of phase synchrony over the whole scalp, and particularly between neighboring electrodes. The use of a corrected measure of synchrony such as the imaginary PLV effectively cancels the contribution of signals with a null or close-to-null phase difference, which is characteristic of two electrodes recording signals from a single common cortical source. The normalized value of the imaginary PLV is comprised between − 1 (when the two signals compared have a constant phase difference of—π/2) and 1 (when the two signals compared have a constant phase difference of π/2). To reject any information about which signal in the pair of electrodes was lagging in phase behind the other, we calculated the absolute value of the imaginary PLV (comprised between 0 and 1) and retained it to estimate interregional synchronization. Such phase information is difficult to interpret due to the cyclicity of the phase and would have  rendered our results more difficult to interpret.

In our analysis, we computed the imaginary PLV between the electrode FC2 (the closest to the stimulation coil targeting the right FEF) and all other scalp EEG leads for the time window [− 500, + 500] ms centered around visual target onset and frequencies between 6 and 50 Hz.

#### Behavioral data analysis

Performance in the detection task was assessed through the perceptual sensitivity index (*d*′), a measure from Signal Detection Theory that quantifies objective perception of stimuli presented around the threshold of perception^46^. Trials were separated in ‘hits’ (when the target was correctly detected), ‘misses’ (when the target was not reported), ‘false alarms’ (when a target was reported for a catch trial, i.e. trials when no target was presented), ‘correct rejections’ (when no target was reported in a catch trial) and ‘errors’ (when a present target was reported on the wrong side of the screen). The perceptual sensitivity index was then calculated from the rate of ‘hits’ and ‘false alarms’ (see ref 30 for full details on such analyses).

#### Statistical analyses

We applied a 2 × 2 orthogonal design with TMS pattern (*rhythmic*, *random*) and TMS condition (*active*, *sham*) as factors. Therefore, values of imaginary PLV, power and ITC were compared in two different ways. First, for each TMS pattern (*rhythmic* and *random*) we contrasted the *active* TMS condition and the *sham* TMS condition. Second, for each of the former two stimulation condition (*active* and *sham*), we compared *rhythmic* TMS and *random* TMS bursts. Pairs of conditions were compared with two-tailed paired Student’s *t*-test (*α* = 0.01). To correct for multiple comparisons in both topographical and time–frequency maps, we performed cluster-based permutation tests with Montecarlo sampling. This method clustered together neighboring electrodes or time–frequency points that reached significance in the paired t-test, using a single *t*-value per cluster. A non-parametric permutation test was then applied on these clusters (10,000 permutations, alpha = 0.05) to determine which ones survived the correction for multiple comparisons. Cluster-based permutations is a highly sensitive method to correct for multiple comparisons in EEG because it is adapted to data highly correlated in space and time (i.e. an effect on the EEG signal is likely to be spread over adjacent EEG leads and consecutive time points)^47^. However, currently no consensus exists on how cluster-based permutations should be applied in factorial designs to evaluate interaction effects between multiple factors^48,49^. For this reason, and guided by our *a piori* predictions of a contrast between *rhythmic* and *random* TMS patterns to isolate the impact of rhythmic structure of pulses within TMS patterns on oscillatory activity, we computed direct pairwise comparisons between conditions.

We initially focused our analyses on EEG signals recorded during stimulation (time window [− 133 0] ms, t=0 being the visual Gabor target onset) and in the high-beta frequency band of interest ([25 35] Hz) and computed the topographical maps for imaginary PLV between electrode FC2 (closest to the targeted right FEF) and all other electrodes on the scalp. However, the localization of significant effects revealed by permutation tests on the topography is not very precise. Indeed, the building of electrode clusters might blur the effect over larger regions. Additionally, it must not be forgotten that the null hypothesis tested in the permutation test in order to control for multiple comparisons extends to the whole array of electrodes and therefore does not permit to fully conclude on the exact localization of any of the observed EEG effects^47^. In order to investigate in further detail the spatial localization of the synchronization induced by *rhythmic* or *random* TMS, we defined two separate regions of interest, one in the left and one in right hemisphere, including parietal and parieto-occipital electrode sites, and compared time–frequency maps of imaginary PLV, Power and ITC for these two regions. All the statistical comparisons of TMS-EEG data were planned as part of an a priori-established strategy testing the hypothesis of increased interregional synchrony under right frontal (FEF) rhythmic high-beta (~ 30 Hz) stimulation within a dorsal fronto-parietal network.

To analyze participants’ performance, a 2 × 2 × 2 repeated measures analysis of variance (ANOVA) was applied to values of perceptual sensitivity index (d′) with *stimulation pattern* (rhythmic, random), *stimulation condition* (active TMS, sham TMS) and *target location* (left, right) as within-participant factors. Planned post-hoc t-student tests were also used for pairwise comparisons (see ref 30 for more details on this analysis).

## Results

### High-beta band right fronto-parietal synchronization

**Figure 2:**
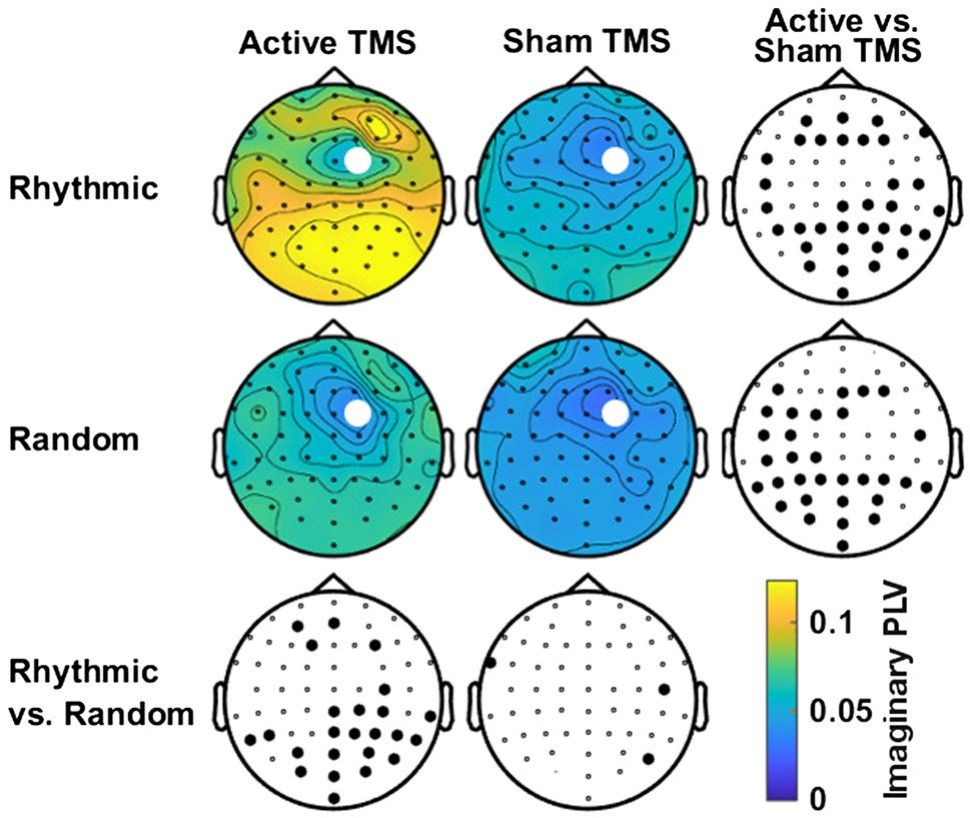
Topographical maps of high-beta imaginary PLV distribution across stimulation patterns delivered to the right FEF. Topographic maps represent imaginary synchrony values (imaginary Phase-Locking Value or imaginary PLV) for each scalp electrode compared to the signal recorded by the electrode closest to the targeted right FEF region and the TMS coil center (10–20 EEG system electrode FC2, signaled with a white dot on the topographic plots), for a time window preceding visual target onset [− 0.133 0] ms and a high beta frequency band [25 35] Hz. Maps are arranged following our 2 × 2 crossed factorial design, comparing *sham* vs. *active* TMS conditions (columns) and *random* vs. *rhythmic* TMS patterns (rows). Bottom and right topographies present the outcomes of the statistical permutation tests, with large black dots indicating EEG electrodes for which imaginary PLV differences reached statistical significance between TMS conditions (p < 0.05), either active/sham rhythmic TMS vs. active/sham random TMS (bottom row) or active rhythmic/random TMS vs. sham rhythmic/random TMS (right column). Note that imaginary synchrony increased significantly in a group of right parietal electrodes for the active *rhythmic* TMS condition compared to both sham *rhythmic* TMS and active *random* TMS control patterns.

Figure 2 illustrates the topographic distribution of imaginary PLV within the high-beta band (25–35 Hz) between contact FC2 (in the scalp EEG array, the closest to the targeted right FEF) and the remaining 59 scalp electrodes, during the TMS delivery period ([− 133 0] ms). Statistical analyses revealed for such frequency band (around the stimulation frequency of *rhythmic *TMS patterns) and time window (TMS burst duration) statistically significant differences between the two types of active TMS patterns. More specifically, *rhythmic* active TMS patterns, compared to *random* active TMS patterns, increased synchronization between the frontal electrode FC2 and a group of leads overlying right parieto-occipital regions ipsilateral to the site of the delivered stimulation (cluster of 21 electrodes, cluster T-stat = 73.717, p-value = 0.0011, Cohen’s d = 0.049). The same pattern of fronto-parietal synchronization was also observed when contrasting active *rhythmic* TMS to sham *rhythmic* TMS bursts (cluster of 25 electrodes, cluster T-stat = 95.882, p-value = 0.0008, Cohen’s d = 0.602).

**Figure 3:**
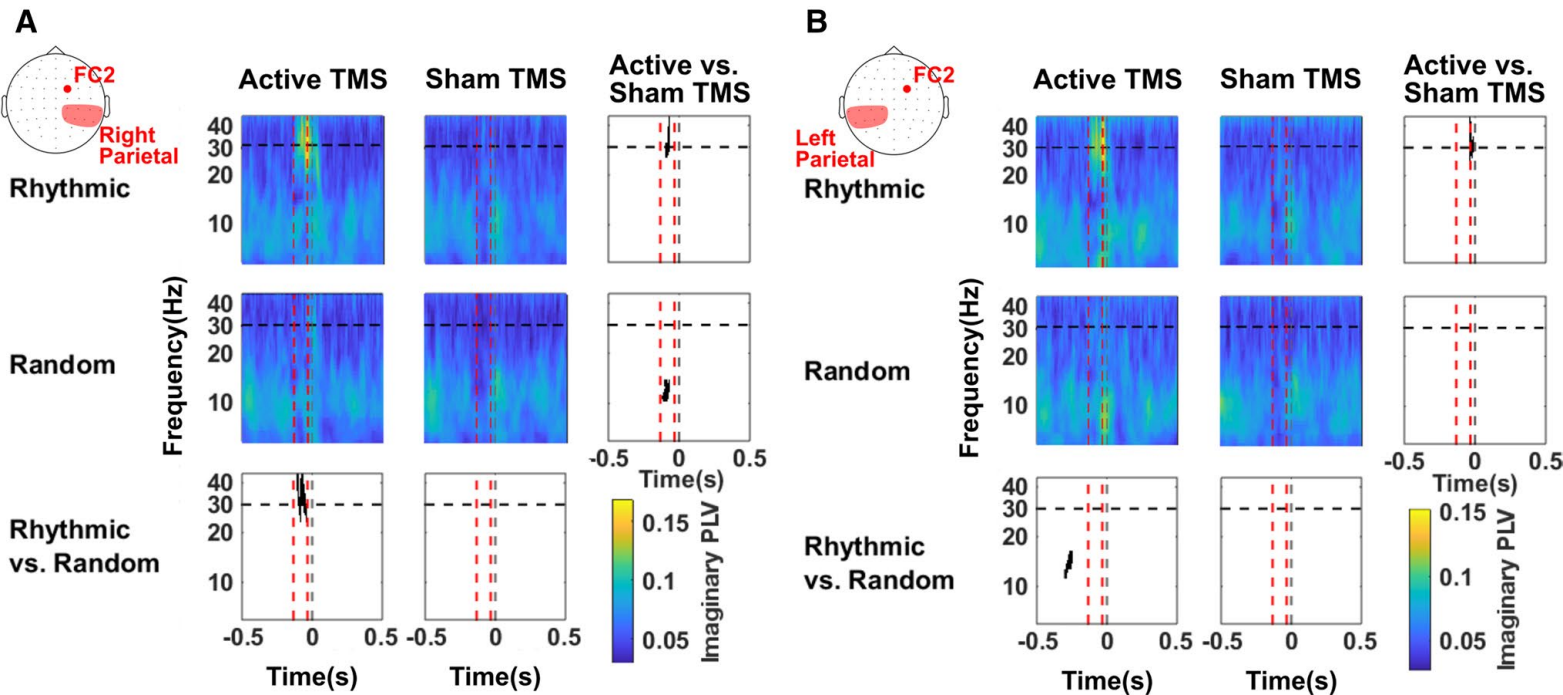
Temporal dynamics and frequency-specificity of fronto-parietal synchronization (imaginary PLV) across stimulation patterns delivered to the right FEF. Time frequency analyses of imaginary Phase-Locking Value (imaginary PLV) between the FC2 scalp EEG electrode and right (**A**) or left (**B**) parietal scalp EEG leads. Time is centered (vertical dotted grey line, t = 0) to the onset of the visual target. Panels are arranged following our 2** × **2 crossed factorial design. We compared *sham* vs. *active* TMS condition (rows) and *random* vs. *rhythmic* TMS patterns (columns). Vertical dotted red lines signal the time window between the 1st (− 133 ms) and the 4th and last (− 33 ms) TMS pulses of the delivered stimulation patterns. The horizontal dotted black line indicates the frequency of TMS rhythmic stimulation pattern (30 Hz). Bottom and right panels present the outcomes of the statistical permutation tests, with black pixels indicating clusters that reached statistical significance between TMS conditions (p < 0.05) either active/sham *rhythmic* vs. active/sham *random* TMS (bottom row) or active rhythmic/random vs. sham rhythmic/random TMS (right column). Note that imaginary fronto-parietal synchrony increased ipsilaterally (right hemisphere) during 30 Hz *rhythmic* stimulation compared to both *random* and sham TMS controls.

Statistical analyses on topographic maps also suggest that *random* TMS patterns (comparison between *random* active and *random* sham TMS conditions) increased synchronization around 30 Hz between the right FEF and fronto-parietal regions in the left hemisphere (contralateral to the stimulation) (cluster of 24 electrodes, cluster T-stat = 90.479, p-value = 0.0001, Cohen’s d = 0.497). Hence, in an attempt to refine the spatial distribution of such increased fronto-parietal synchronization observed in response to *rhythmic* or *random* TMS, we defined two regions of interest comprised of left or right parietal and parieto-occipital scalp EEG contacts. Statistical analyses on time–frequency datasets (Fig. 3) confirmed that, compared to *random* TMS bursts, *rhythmic* TMS patterns increased right fronto-parietal synchrony only during the delivery of active stimulation and that such effects operated within a frequency band restricted to high-beta (24–45 Hz) oscillations (Fig. 3A)(cluster T-stat = 279.485, p-value = 0.001, Cohen’s d = 1.235). A similar result emerged when comparing the *rhythmic* active and sham conditions (cluster T-stat = 98.341, p-value = 0.023, Cohen’s d = 1.177). However, contrary to what was suggested by our first topographic analysis, no statistically significant effects were found for active *random* TMS patterns for left fronto-parietal synchrony (Fig. 3B comparison between *random* active and *random *sham TMS conditions).

Topographic and time–frequency analyses on PLV between frontal and parietal scalp EEG contacts suggested that the delivery of *rhythmic* TMS patterns over the right FEF induced an increase of inter-regional synchronization in the high-beta band. Such increases proved short-lasting (i.e., not exceeding the duration of 4 pulse TMS bursts ~100 ms) and were likely entrained across a fronto-parietal network of cortical sites within the targeted right cerebral hemisphere.

### Long-distance oscillatory parietal entrainment of high-beta band activity

**Figure 4:**
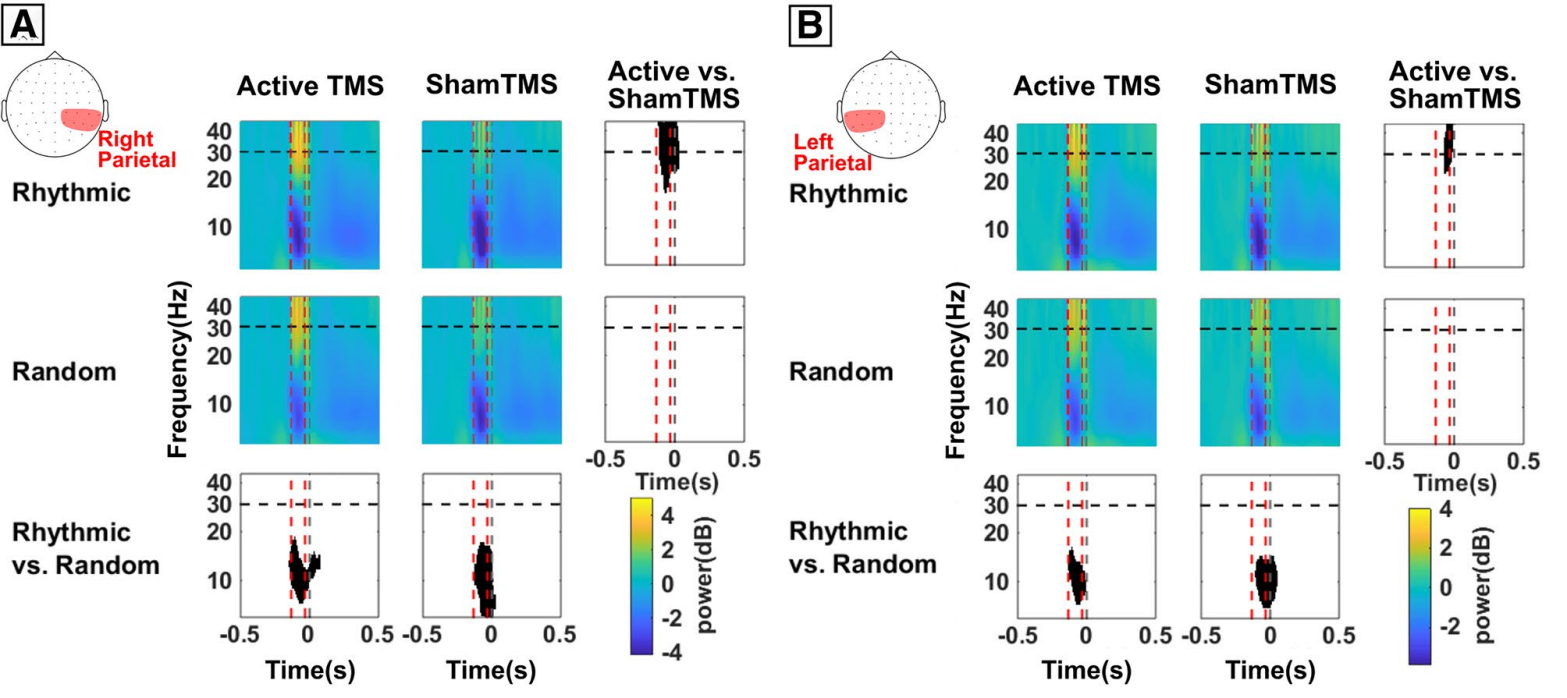
Temporal dynamics and frequency-specificity of high beta local entrainment (power) on parietal regions across stimulation patterns delivered to the right FEF. Time frequency plots representing oscillation power over right parietal (**A**) or left parietal (**B**) electrodes. Time frequency panels are arranged following a 2** × **2 crossed factorial design, in which we compared *sham* vs. *active* TMS condition (rows) and *random* vs. *rhythmic* TMS patterns (columns). Time is centered on the onset of the visual target (vertical dotted grey line, t = 0). Vertical dotted red lines indicate the time window between the 1st (− 133 ms) and the 4th and last (− 33 ms) TMS pulses of the delivered stimulation pattern. The horizontal dotted black line indicates the delivered frequency of stimulation (30 Hz) for rhythmic patterns. Bottom and right panels present the outcomes of the statistical permutation tests, with black pixels indicating clusters that reached statistical significance between TMS conditions (p < 0.05), either active/sham *rhythmic* vs active/sham *random* (bottom row) or active rhythmic/random vs. sham rhythmic/random (right column). Notice that right frontal active *rhythmic* stimulation, compared to sham stimulation, transiently increased high-beta oscillation power over the left and right parietal cortices, hence at distance from the stimulation site. However, no differences were found for the time course of 30 Hz power comparing active *rhythmic* vs. active *random* TMS patterns. Note also that the sound of the TMS coil delivering rhythmic stimulation might be responsible for the decrease (desynchronization) of alpha oscillations in bilateral parietal regions found in the four conditions.

Oscillation power within the 25–35 Hz high-beta band in both left and right regions of interest (i.e., parietal and parieto-occipital EEG contacts) increased significantly in response to active *rhythmic* TMS patterns delivered frontally, compared to sham *rhythmic* TMS bursts (right hemisphere: cluster T-stat = 1084.6, p-value = 0.0016, Cohen’s d = 1.197; left hemisphere: cluster T-stat = 337.3, p-value = 0.0136, Cohen’s d = 1.125), but not during active *random* frontal stimulation, compared to equivalent sham TMS bursts (Fig. 4).

**Figure 5:**
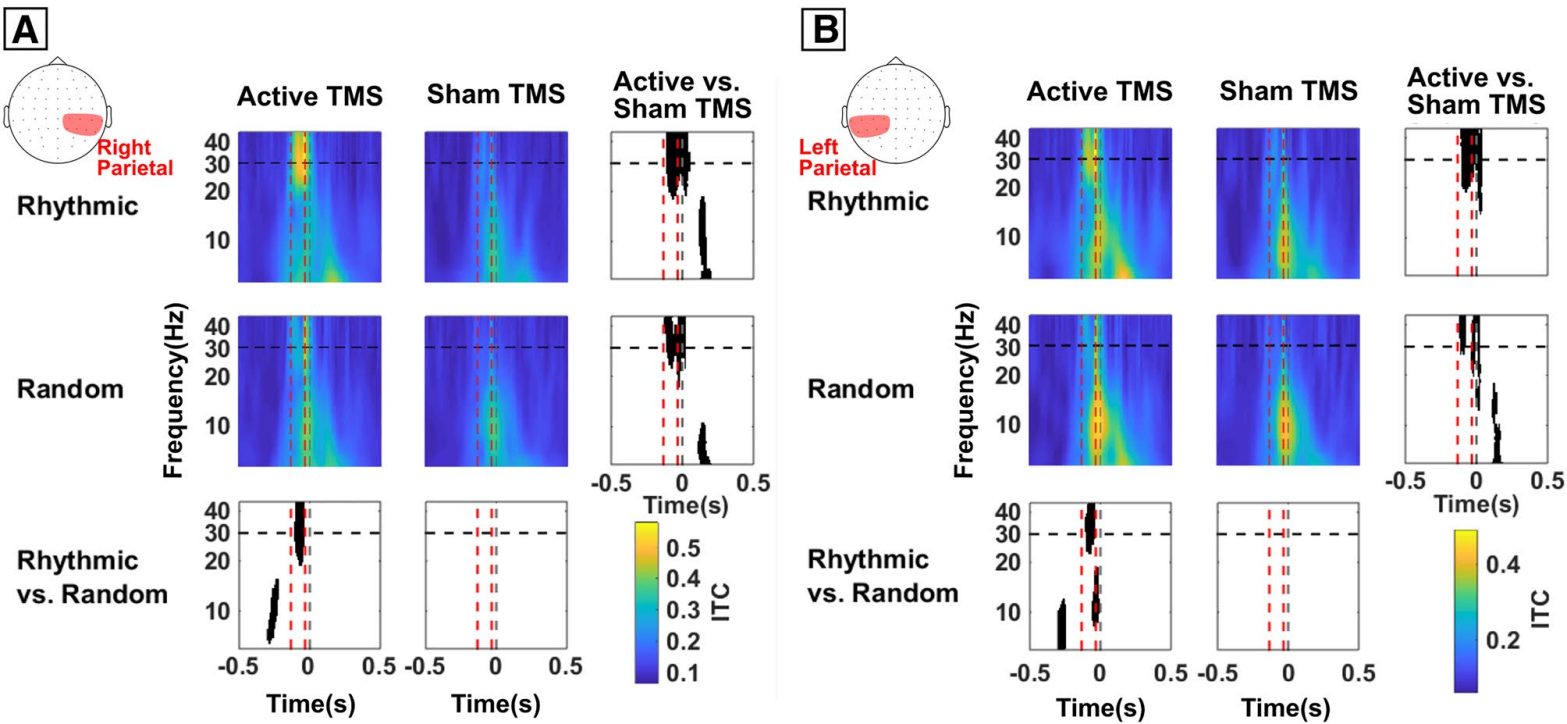
Temporal dynamics and frequency-specificity of distant phase-locking of high beta oscillations on parietal regions across stimulation patterns delivered to the right FEF. Time frequency plot of inter-trial coherence (ITC) over right parietal (**A**) and left parietal (**B**) scalp EEG electrodes. Time frequency panels are arranged following a 2 × 2 crossed factorial design, in which we compared *sham* vs. *active* TMS condition (rows) and *random* vs. *rhythmic* TMS patterns (columns). Time is centered on the onset of the target (vertical dotted grey line, t = 0). Vertical dotted red lines indicate the time window between the 1st (− 133 ms) and the 4th and last (− 33 ms) TMS pulses of the delivered stimulation pattern. The horizontal dotted black line indicates the frequency of rhythmic TMS stimulation (30 Hz). Bottom and right panels present the outcomes of the statistical permutation tests, with black pixels indicating clusters that reached statistical significance between TMS conditions (p < 0.05), either active/sham rhythmic vs active/sham random (bottom row) or active rhythmic/random vs. sham rhythmic/random (right column). Note that *rhythmic* stimulation of the right FEF increased inter-trial coherence in the high-beta range distantly over right and left parietal EEG contacts, whereas *random* stimulation patterns increased phase-locking transiently in the alpha frequencies in parietal EEG leads contralateral to the site of right FEF stimulation.

Inter-trial coherence (ITC), a measure of phase-locking of local oscillations, was also found to be significantly increased in left and right posterior parietal regions of interest following active *rhythmic* TMS to the right FEF (Fig. 5). Indeed, the comparison between active and sham TMS conditions showed higher ITC levels during active stimulation for both *rhythmic* (Right hemisphere: cluster T-stat = 1710.3, p-value = 0.0001, Cohen’s d = 1.783; Left hemisphere: cluster T-stat = 1502.4, p-value = 0.0002, Cohen’s d = 2.409) and *random* (Right hemisphere: cluster T-stat = 467.9, p-value = 0.0029, Cohen’s d = 1.441; Left hemisphere: cluster T-stat = 396.8, p-value = 0.0067, Cohen’s d = 1.804) TMS patterns in bilateral parietal sites. However, a direct comparison between the two active conditions (*rhythmic* vs. *random* TMS patterns) revealed higher levels of a trial-by-trial phase-locking for *rhythmic* TMS patterns compared to *random* TMS patterns (Right hemisphere: cluster T-stat = 276.2, p-value = 0.0194, Cohen’s d = 1.202; Left hemisphere: cluster T-stat = 452.6, p-value = 0.0036, Cohen’s d = 1.498).

### Rhythmic and random TMS effects in alpha band synchronization

Aside from the modulation of oscillatory activity in the high-beta band, our data also unexpectedly revealed signs of alpha desynchronization over parietal areas for all  four TMS conditions (active or sham, *rhythmic* and *random* TMS stimulation) during burst delivery (Fig. 4). This outcome might not be likely associated to TMS active fields, as the delivered bursts failed to show any significant difference when comparing active vs sham TMS patterns. However, both statistical *rhythmic*/*random* comparisons (active *rhythmic* vs. active *random* and also sham *rhythmic* vs. sham *random*) showed that *rhythmic* patterns induced stronger alpha desynchronization than *random* patterns (active *rhythmic* vs. *random*, right hemisphere: cluster T-stat = − 716.70, p-value = 0.0045, Cohen’s d = 1.325, left hemisphere: cluster T-stat = − 483.5, p-value = 0.0073, Cohen’s d = 1.138; sham *rhythmic* vs. *random*, right hemisphere: cluster T-stat = − 1026.1, p-value = 0.0037, Cohen’s d = 1.498, left hemisphere: cluster T-stat = − 929.1, p-value = 0.002, Cohen’s d = 1.25).

By the end of the TMS burst, we observed alpha band phase-locking increases over the same parietal contacts (Fig. 5). Although visible in all four conditions (note that, again, this phase-locking was not significantly different between active and sham TMS patterns), phase-locking in left parietal contacts (hence on the hemisphere contralateral to the stimulation) was this time stronger for the active *random* stimulation compared to active *rhythmic* stimulation (Fig. 5B). Further investigations with a more adapted behavioural paradigm will be necessary to better pinpoint the origin and potential contribution of such alpha band activity.

### Effect of high-beta synchronization on conscious visual detection

As previously published in a study performed on the same dataset we further ﻿analyzed here^30^, *rhythmic* stimulation at 30 Hz delivered on the right FEF modulated the outcomes of a visual detection task performed by participants. Briefly, a 2 × 2 × 2 ANOVA on perceptual sensitivity (d’) values revealed a main effect of *stimulation condition* (active or sham) (F(1,13) = 5.33; p < 0.05), with higher levels of visual sensitivity (d′), i.e. better detection performance, in trials in which the right FEF had been stimulated with active TMS, but indistinctively for *rhythmic* and *random* TMS patterns.

Statistical analyses also revealed a significant main effect of *visual field* (right, left) (F(1,13) = 10.14; p < 0.01) with higher perceptual sensitivity (d′) for right visual targets compared to those presented in the left visual hemifield. No other significant effects were found, although a three-way interaction *visual field* x *stimulation pattern* x *stimulation condition* displayed a trend towards statistical significance (F(1,13) = 3.97; p < 0.068).

**Figure 6:**
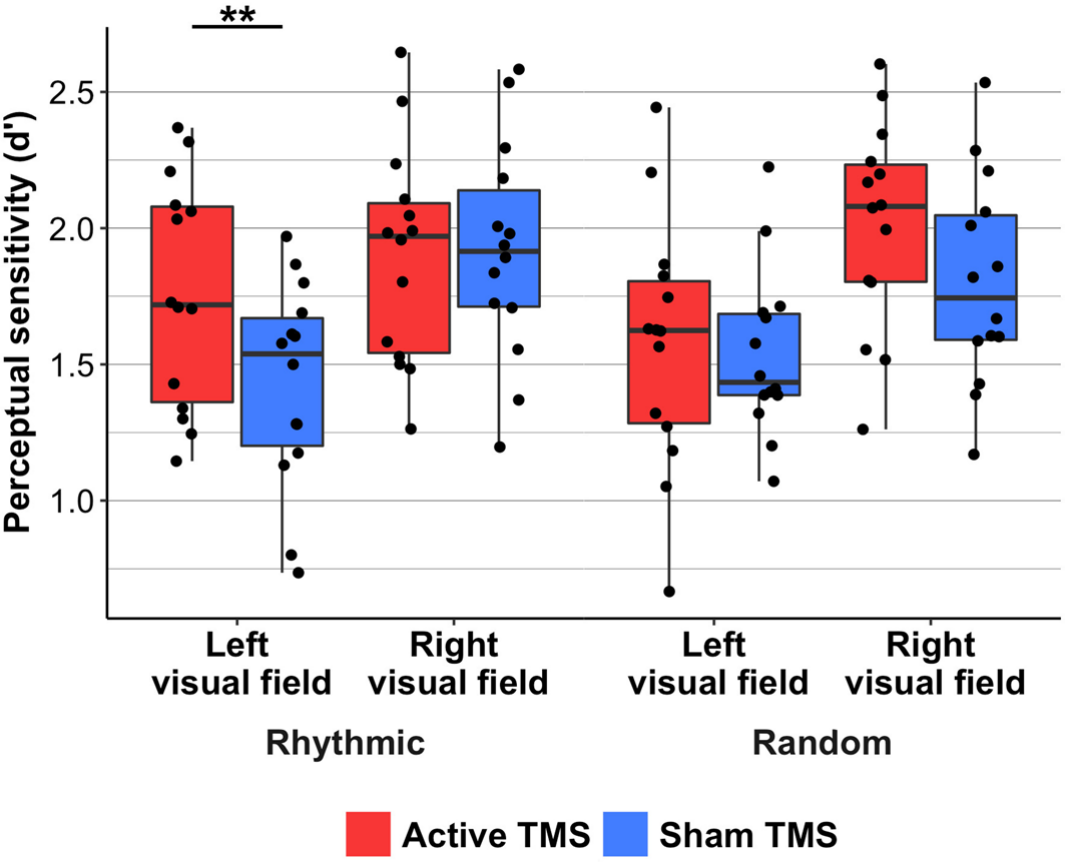
Modulation of perceptual sensitivity (d**′**) for left and right hemifield visual targets presented across TMS stimulation patterns delivered to the right FEF. Boxplots are presented for the rhythmic (left) and random (right) stimulation conditions contrasting active (in red) vs sham (in blue) patterns for targets presented in the left (contralateral to right FEF stimulation) and the right (ipsilateral to right FEF stimulation) visual hemifield. Boxes are drawn from the 25th to the 75th percentile and the horizontal line indicates the median. Whiskers are drawn between the minimum and maximum data points. Black dots  represent individual d**′** measures for each individual participant (for details on statistical approaches refer to the Results section). ** : p < 0.01 in post-hoc t-tests between active vs sham conditions. Notice that perceptual sensitivity was increased only for targets contralateral to the stimulation site (left) in active compared to sham *rhythmic* TMS patterns. Decision criterion (not presented in the current manuscript) was not significantly modulated by differnt patterns of stimulation. Note also that the current figure, showing the behavioural impact on conscious visual detection across different TMS conditions has been compiled with a dataset published previously^30^ in different graphic form.

On the basis of a strong a priori hypothesis supporting different effects for *rhythmic* and *random* stimulation patterns on conscious visual perception^18,34,36^ (see ref 30 for further details on statistical analyses for visual outcomes), Student’s t-test were applied to compare active vs. sham TMS patterns. These analyses revealed that *rhythmic* active TMS (compared to its equivalent sham TMS) increased perceptual sensitivity (d′) for targets displayed on the left visual field (p < 0.01) but not for those presented on the right (p > 0.88) (Fig. 6). No significant differences were found between active *random* and sham *random* TMS patterns, neither for right nor for left targets (both active vs. sham comparisons p > 0.11).

## Discussion

Here we used rhythmic TMS coupled to EEG recordings during a conscious visual detection paradigm to explore in humans the contributions of 30 Hz inter-regional synchrony across right dorsal fronto-parietal systems and assess their potential causal implications in the modulation of conscious visual perception. Our data suggests that high-beta rhythmic TMS patterns delivered over the right FEF induced synchronization in the high-beta band between this frontal area and activity recorded over ipsilateral parietal contacts. This increase in synchronization was transient and did not extend beyond the delivery of the last pulse of the rhythmic TMS burst. In our manuscript, phase-synchronization measures were carefully corrected (by using imaginary PLV as a measure of interregional phase coupling) to ensure that increases in phase-synchronization recorded at the scalp level stemmed from inter-regional synchronization between two distinct cortical sources. Since synchronization increases were not observed when participants were stimulated with our control *random* TMS pattern (made of an equal number of pulses and total duration as *rhythmic* 30 Hz patterns), we conclude that this effect is dependent on the specific spatio-temporal structure carried by our high-beta *rhythmic* pattern. We also posit that, very likely, such effects on fronto-parietal synchrony are closely related to the local entrainment of a 30 Hz episodic rhythm induced by TMS in the right FEF characterized in a prior publication^30^.

Although not the focus of the current study, some level of skepticism remains with regards to the ability to dissociate oscillatory entrainment from a simple repetition of evoked potentials induced by series of individual periodical stimulation pulses. Early references in the field stated that genuine entrainment must be characterized by a progressive build-up of oscillation amplitude over the course of the TMS bursts; whereas in contrast, repeated evoked potentials would exhibit constant amplitude^26^. To this regard, prior analyses of this same dataset focusing on local right FEF entrainment phenomena contributed evidence of a progressive increase of high-beta evoked oscillations during 30 Hz rhythmic TMS on right frontal regions. Such data yielded support in favor of oscillatory entrainment during stimulation as opposed to a set of rhythmic activity arising from individual evoked potentials, repeating at the stimulation frequency^30^. Nonetheless, additional research might still be needed to identify new criteria which allow an accurate differentiation of these two types of events.

Given that focal stimulation has been shown to combine local and inter-regional effects^24,25^ we here investigated if the focal entrainment of a 30 Hz oscillation in right frontal regions could be conveyed to distant interconnected cortical regions, not directly influenced by TMS patterns. To this end, we focused our analyses on EEG leads overlying posterior parietal regions, notably those located over and around the intraparietal sulcus (IPS), which has been shown to interact with the right FEF as part of the dorsal attentional orienting network and shown an ability to modulate the visibility or saliency of perceptual inputs^32–34^. As initially predicted, our analyses revealed that 30 Hz rhythmic TMS patterns delivered to the right FEF did not only entrain, as shown previously, right frontal high-beta activity^30^ but increased high-beta power and phase-locked high-beta oscillations over posterior parietal electrodes.

This phenomenon could be likely explained by the entrainment of cortical oscillations via episodic TMS bursts delivered to a distant but richly interconnected area^26^. However, our data suggests that phase synchronization (assessed by increases of imaginary PLV) increased only for right EEG contacts, whereas signs of parietal entrainment (assessed with increases of ITC) was recorded in both right and left parietal electrodes. Accordingly, we cannot rule out the possibility that posterior parietal entrainment may be partially independent from direct intrahemispheric fronto-parietal synchronization mechanisms and might have engaged other interregional interactions.

The electrophysiological effects induced by rhythmic TMS bursts delivered shortly before the presentation of a low contrast Gabor target were accompanied by modulations of conscious visual performance, consisting in increases of perceptual sensitivity (d’)^30^. Taken together, our results contribute evidence towards an implication of high-beta oscillatory activity, operating across fronto-parietal systems (and potentially involved in the allocation of visuo-spatial attention), in the modulation of conscious visual detection performance.

### Fronto-parietal brain rhythms and the modulation of attention and perception

Our results are consistent with influential findings by Buschman and Miller^13^ in non-human primates, obtained by means of intracranial recordings and providing correlational evidence in favour of high-beta fronto-parietal synchrony during the allocation of endogenous attention in a top-down visual search task. Such work was replicated in humans employing scalp EEG^50^. Our study used a simple conscious detection task which did not directly manipulated the orienting of visual attention by means of spatial or attentional cues. Nonetheless, this modality of attention might have been engaged in our participants since, on each trial, they were informed with an central alerting cue (fixation cross increasig its size) about the onset of an upcoming lateralized target within a given delay (~ 233 ms) for which only endogenous attention can uphold expectancy^51^.

Our results build on the above-mentioned correlational results and, adding the value of manipulating brain rhythms with different TMS patterns, provide further evidence suggesting a potential causal relationship between high-beta (~30 Hz) fronto-parietal synchrony and the modulation of visual perception in the human brain. Most importantly, our work extends prior results derived from this same TMS-EEG dataset and suggests that short bursts of focal rhythmic TMS do not only show an ability to locally entrain frequency-specific rhythms dictated by the pace of stimulation pulses, within the targeted cortical area^30^ but can also be used to synchronize in a frequency-specific manner the stimulated target region (in our case the right FEF) with interconnected areas (such as posterior parietal sites) and entrain distantly rhythms at this same frequency.

Last but not least, our analyses also support the suitability of *rhythmic* TMS patterns delivered onto a specific cortical location to isolate the contribution of oscillation frequency to cognitive functions and behaviours, by comparing   the impact on task performance under *rhythmic* TMS vs. control *random* patterns (of equal duration and number of pulses). Owing to the use of TMS *random* patterns as a control condition, the here reported TMS-driven electrophysiological EEG differences between active *rhythmic* and *random* TMS conditions are unlikely to be artefacts caused by the auditory (clicking) or tactile (scalp tapping) stimulation inherent to the delivery of TMS pulses. Neither could they be simply explained by the impact of magnetic pulses on neural activity, since in that case potential artefactual effects of single pulses would be identically present in both active TMS conditions, *rhythmic* and *random*.

Pioneering research in this field has extensively employed rhythmic TMS (without coupled EEG recording) to investigate the causal role of local oscillatory activity on different cognitive processes subtending behavioural performance^20,28,29^. Nonetheless, the current report is among the first to use coupled online TMS-EEG recordings to gather evidence suggesting a causal link between a complex cognitive process such as the modulation of conscious perception subtended by long-range cortical systems (such as the dorsal attention orienting networks) and specific modes of interregional synchronization between frontal and posterior parietal brain regions. In any case, further studies combining the direct and specific manipulation of fronto-parietal oscillatory synchronization per se, for example by means of multi-coil TMS approaches^52^, during the assessment of visual perception abilities would be required to strengthen and further confirm the current results.

### Causal role of inter-regional synchrony in visual perception improvements

The detailed mechanistic explanations for our electrophysiological effect and its associated behavioural findings remain open. Nonetheless in their influential non-human primate study^13^, Buschman and Miller hypothesized that synchronization of neuronal activity may increase the efficiency of inter-areal coordination and communication, enabling to process a single object and to suppress distractors. This hypothesis is consistent with the explanatory model developed in 2009 by Fries, in which inter-regional synchronization in the gamma-band was proposed to provide an exclusive and effective communication link between two areas selective for a visual target and invariant even in the presence of distractors^9^.

The limited spatial resolution of scalp EEG (even more limited with the use of 60 EEG electrode grids) does not allow us to pinpoint with high anatomical precision which specific cortical regions were synchronized during right frontal TMS *rhythmic* stimulation. Nonetheless, the cortical focality provided by TMS, targeting (guided by means of a MRI-based frameless stereotaxic neuronavigation system) the Tailarach coordinates of the right FEF allows at least to put forward a prediction regarding the localization of cortical sources of fronto-parietal synchrony. Indeed, coherent with the spatial resolution of TMS, prior reports and published data by our group make plausible to hypothesize that high-beta cortical oscillations were entrained primarily on the TMS target, the right FEF^26,30^, and neighboring sites. The FEF is anatomically linked to the posterior parietal intraparietal sulcus (IPS) via a white matter tract known as the 1st branch the Superior Longitudinal Fasciculus (SLF I)^52^. According to prior correlational work^53,54^ and causal evidence^18^, the system defined between areas linked by this major white matter bundle, the so called dorsal attentional network, plays a major role in the allocation of visuo-spatial attention and the top-down modulation of visual perception.

Additionally, prior reports employing tensor diffusion imaging in participants who underwent non-invasive stimulation combined with perceptual tasks identical to those used in the current study^33,34^ showed that TMS-driven improvements of visual detection scaled significantly with anatomical features of the SLF I white matter bundle, hence supporting the hypothesis that fronto-parietal synchronization might operate along this specific tract. In sum, we here propose that local entrainment of oscillatory activity in a high beta frequency (~ 30 Hz) impacted the right FEF and may have presumably spread, through white matter projections of the SLF I, between the right frontal and posterior parietal regions, encompassing, respectively, the FEF and the IPS. Moreover, this white-matter tract could underlie frequency-specific synchronization between areas of the dorsal attention orienting network responsible for enabling spatial attention, and by virtue of such effect, subserve more efficient communication between right frontal and posterior parietal sites. More specifically, a hypothesis which would be worth testing in future studies is that increased synchronization may be beneficial for a fast and flexible allocation of spatial attention, enhancing visual sensitivity and facilitating access to visual consciousness.

In any case, further studies employing electrophysiological recordings and stimulation techniques with higher spatial resolution, or additional TMS experiments specifically targeting the right IPS, would be needed to further pinpoint the precise anatomical substrate subtending high-beta oscillations and their potential role driving improvements of conscious visual perception.

The right fronto-parietal localization of our effects could be predicted on the basis of prior evidence showing a significant correlation between the volume of the SLF I and TMS-induced improvements of visual perception^33,34^. Nonetheless, we decided not to constrain our ad hoc analyses according to any *priori* prediction on the loci potentially involved in high-beta synchronization. Instead, we computed measures of synchrony between the closest electrode overlaying the right FEF and all scalp EEG leads out of a full array of 60 EEG contacts. It is hence rather remarkable that our statistical analyses revealed a significant synchronization, tied to the duration of the delivered rhythmic TMS patterns, between the right FEF and right parietal electrodes.

### Modulation of alpha oscillations

In addition to the modulation of high-beta oscillations, the implemented TMS manipulation showed effects on alpha band oscillations (Figs. 4 and 5). Since these modulations were present in active and also sham TMS conditions, we hypothesize that they did not emerge from a direct manipulation of neural activity by TMS pulses, and could instead be explained by an effect of the clicking sound accompanying the delivery of TMS pulses. The loud sound accompanying the delivery of active or sham TMS bursts, present in all four TMS conditions 133 ms before visual target onset (in *rhythmic* or *random* bursts), could have had an alerting effect, prompting participants to engage sustained or selective attention mechanisms to focus on the task and the computer screen. The role of alpha desynchronization as a marker of attentional orienting has been well established^32,53^ hence, the strong alpha desynchronization observed in all four TMS tested conditions could be consistent with this proposition.

Alternatively, the phase-locking of alpha oscillations observed after the last TMS pulse could have emerged as an evoked effect of the loud TMS clicking sound, through a different mechanism. Indeed, sounds have been found to cross-modally phase-lock alpha oscillation in occipital cortices^54^. Hence, an interaction between cross-modal alpha phase-locking and random TMS patterns engineered to entrain a wide range of frequencies (amongst them alpha) on different trials, could explain significantly higher alpha phase-locking over the left parietal region for active *random* compared to active *rhythmic* TMS conditions. Much remains to be understood about these modulations in the alpha range. However, as these were not predicted, hence outside of the initial focus of this article, it will be the role of further studies with better adapted tasks to shed more light onto these phenomena.

## Conclusions and future directions

At the methodological and technological level, the current study supports our ability to manipulate ‘at will’ inter-areal network synchrony using mono-focal rhythmic TMS delivered to single cortical region and sets the stage for promising future uses of this same approach to systematically map between new sets of cortical sites,  synchrony interactions across frequency bands, underlying other cognitive processes, behaviours and their dysfunctions. At the fundamental level, our findings extend prior evidence derived from correlational approaches and invasive electrophysiological recordings in non-human primates. To this regard, (1) they add causality to suspected associations between right fronto-parietal synchronization in the high-beta band and the modulation of conscious visual performance in human participants; (2) They contribute solid evidence to support a role for high-beta right frontal oscillations and fronto-parietal synchrony as relevant physiological coding strategies for top-down strategies to modulate visual perception; Important for the field of brain plasticity, (3) they set the stage for future applications of focal non-invasive rhythmic stimulation (via TMS or other transcranial stimulation technologies, such as transcranial Alternate Current Stimulation (tACS) or Focused Ultrasound Stimulation (FUS)) to modulate local and inter-regional synchrony, in order to facilitate cognitive performance in healthy participants or improve dysfunctional synchrony patterns subtending neuropsychiatric conditions in patients.

## Supplementary information

Supplementary Information 1.

## Data Availability

Data are available from the corresponding author upon request.
