# Supplementary material for: Causal modulation of right hemisphere fronto-parietal phase synchrony with Transcranial Magnetic Stimulation during a conscious visual detection task
Source: Sci Rep. 2021 Feb 15;11:3807. doi: 10.1038/s41598-020-79812-y (PMC7884390; doi:10.1038/s41598-020-79812-y)
Supplement: Supplementary file 1 — Supplementary Information 1. [file 41598_2020_79812_MOESM1_ESM.pdf]

Causal modulation of right hemisphere fronto-parietal phase synchrony with Transcranial Magnetic Stimulation during a conscious visual detection task

Chloé Stengel[1], Marine Vernet[1,2], Julià L. Amengual[3], and Antoni Valero-Cabré[1,4,5*]

[1] Cerebral Dynamics, Plasticity and Rehabilitation Group, FRONTLAB team, Institut du Cerveau et la Moelle Épinière (ICM), Pitié-Salpêtrière Hospital, CNRS UMR 7225, INSERM U 1127 & Sorbonne Université, 47 boulevard de l'Hôpital, Paris 75013, France

[2] IMPACT team, Lyon Neuroscience Research Center (CRNL), CNRS UMR 5292, INSERM UMRS 1028, University Claude Bernard Lyon 1, Lyon, France

[3] Institut des Sciences Cognitives Marc Jeannerod, CNRS UMR 5229 and Université Claude Bernard Lyon 1, Lyon, France

[4] Laboratory for Cerebral Dynamics Plasticity and Rehabilitation, Boston University, School of Medicine, Boston, MA, USA

[5] Cognitive Neuroscience and Information Technology Research Program, Open University of Catalonia (UOC), Barcelona, Spain

Supplementary Methods

1. Control Analyses to assess the influence of TMS artifact removal and EEG data cleaning

We conducted a control analysis which aimed at confirming that our TMS-EEG artifact removal and cleaning method was adequate and that the presence of rhythmically-spaced TMS artifacts did not artificially increase, compared to randomly-spaced TMS artifacts, estimates of high-beta oscillatory activity employed in our study such as: Power, Inter-Trial Coherence (ITC) and Inter-regional Phase Synchronization (imaginary PLV or imPLV).

To this end, a large sample of waveforms of individual TMS artifacts were extracted from 150 randomly selected trials from each of our two collections of 'real' active *rhythmic* and *random* TMS-EEG datasets recorded across all participants during the current study. Individual TMS artifacts were detected on single trials using the automated artifact detection algorithm implemented in the FieldTrip toolbox on MATLAB (Mathworks). Data were pre-processed to maximize 'jump artifacts', and the envelope of the signals was z-normalized. Any data sample that crossed a z- value threshold of 2.4 was considered part of a TMS-artifact (see Figure S1B, left panel, signal in red). TMS artifacts were then extracted from 'real' active TMS-EEG datasets and added to epochs of 'real' eyes-open resting-state EEG datasets obtained from the same cohort of healthy participants (Figure S1B).

This procedure was applied to both *rhythmic* and *random* TMS-EEG traces, and resulted in a set of n=300 'artificially artifacted' real resting-state EEG trials, in which half of them contained rhythmically-spaced TMS artifacts (at a 30 Hz frequency), while the other half contained randomly-spaced TMS artifacts (i.e., implementing the timings of artifacts present in 'real' active *random* TMS-EEG datasets). Then, 'artificially artifacted' EEG datasets underwent the same artifact removal and cleaning procedure than the 'real' active and sham TMS-EEG data presented in our study. Briefly, signal within a a [-4 +12] ms window around each TMS pulse onset was eliminated and then filled in with signal interpolation. An ICA was then performed to eliminate

remaining TMS artifacts as well as eye movements, electrode malfunction and 50 Hz land noise artifacts.

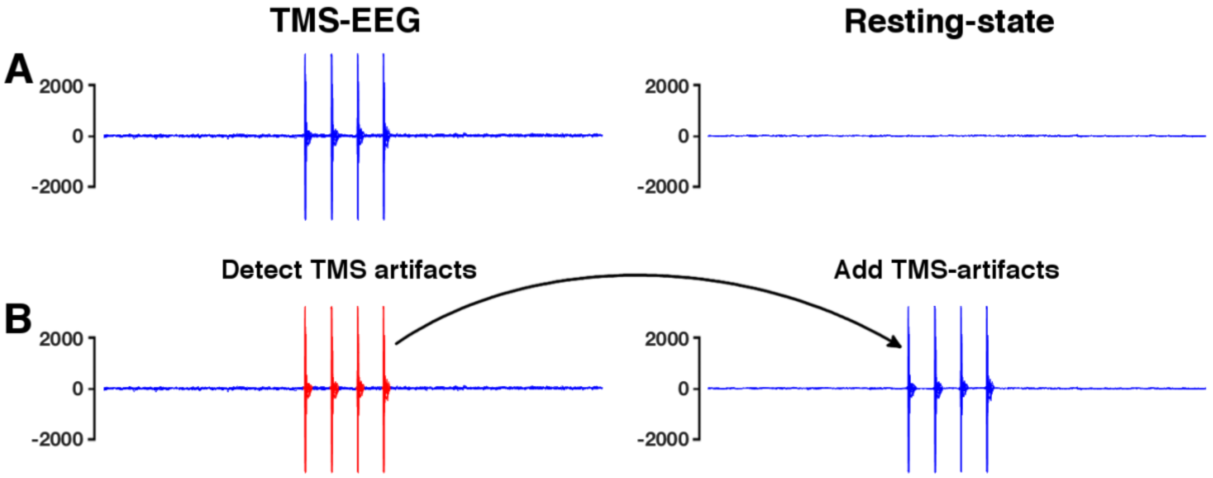

Figure S1: Control analysis to address the influence of TMS artifact and TMS-EEG cleaning procedures on oscillatory activity. The figure shows selected representative EEG traces of an individual participant for the following conditions: **(A**, left & right**)** 'real' active TMS-EEG datasets and 'real' eyes-open resting state EEG datasets. **(B)** Addition of TMS-artifacts from randomly selected 'real' active TMS-EEG trials (left) to 'real' eyes-open resting-state EEG datasets (right).

After data cleaning, we computed values of power, ITC and imaginary PLV, identical to those described in the main text, on both datasets (*rhythmic* and *random* 'artificially artifacted' real resting-state EEG data). Spectral power was computed for each individual trial by applying the same wavelet-based methods described in the main article. We then compared measures of Power, ITC and imPLV in the high-beta band ([25 35] Hz) during *rhythmic* and *random* 'artificially artifacted' periods (time window of stimulation: [-133 0] ms).

Topographies of high-beta power were compared within trials with paired Students t-test ($\alpha = 0.01$). A cluster-based permutation test with Monte Carlo sampling was performed to correct for multiple comparisons (10000 permutations, $\alpha = 0.05$). For ITC and imPLV we were unable to apply the same statistical procedure because values of ITC and imPLV were not available for individual trials (both measures require averaging of phase or phase-difference over all trials) and therefore within-trials t-tests could not be performed. Hence for these two outcome measures, we applied cluster-based permutation tests on the difference of ITC and imPLV between the two

compared conditions. A null distribution was created by computing the difference in ITC and imPLV from two groups of trials with their labels randomly permuted 10000 times (i.e., for 10000 permutations). The true difference in ITC and imPLV in unpermuted trials was then compared to this random distribution. Any cluster of electrodes that exceeded the significance threshold of α = 0.05 were reported as showing significant differences between the compared conditions.

Statistical results show that none of the high beta power (Figure S2A), ITC (Figure S2B) or ImPLV (Figure S2C) measures resulted in statistically significant differences between *rhythmic* and *random* ‘artificially artifacted’ EEG data. This indicates that the rhythmic or periodical structure of stimulation artifacts does not modulate outcome measures estimating oscillation amplitude (Power) or phase-locking (ITC and imPLV).

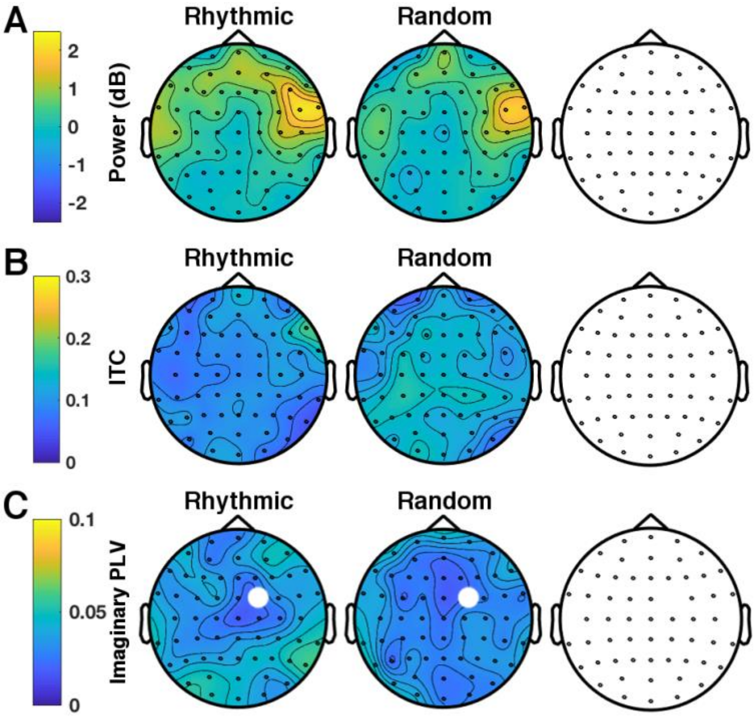

Figure S2: Control Analysis to assess the impact of the rhythmic structure of TMS artifacts on estimates of oscillatory activity. Topographic maps represent **(A)** oscillation power, **(B)** Inter-trial coherence (ITC) and **(C)** imaginary synchrony values (Imaginary Phase-Locking Value, imPLV) for high-beta oscillations ([25 35] Hz) during the TMS stimulation window ([-0.133 0] ms) for rhythmic and random ‘artificially artifacted’ resting-state EEG data. **(C)** Imaginary synchrony values were computed for each scalp electrode relative to signals recorded by contact FC2 (labelled with a white dot, located closest to the TMS coil center). Right topographies present the outcomes of statistical permutation tests for the comparison of *rhythmic* and *random* ‘artificially artifacted’ EEG data. Notice that none of the contacts showed significant differences for any of the comparisons.

2. Control Analyses to dissociate oscillatory entrainment and rhythmically repeated TEPs

In order to provide evidence for genuine oscillatory entrainment during rhythmic stimulation, as opposed to single pulse TMS-evoked activity repeated at a 30 Hz frequency, we conducted a second control analysis that aimed to demonstrate that increases in high-beta inter-trial coherence observed in our data were not caused by a succession of single pulse evoked responses with constant amplitude.

To this end, we generated synthetic data to simulate the repetition of single pulse evoked response. We considered that the data that most closely emulated TEPs produced by single TMS pulses was the segment of EEG signals directly following the 1st pulse of each TMS bursts from our rhythmic TMS-EEG dataset. Therefore, we extracted epochs of EEG data immediately preceding the 1st pulse of each rhythmic TMS burst to a few milliseconds before the onset of the 2nd pulse (window of [-3 +30] ms centered around 1st pulse onset). We did so for all electrodes, trials and participants of our cohort (n=14) in our *rhythmic* TMS-EEG dataset. This extracted epoch was repeated 4 times in order to create a set of 'synthetic' data for each participant that simulated a burst of identical TMS evoked responses repeated at a 30 Hz frequency (Fig S3A).

Measures of inter-trial coherence (ITC) and imaginary Phase-Locking Value (imPLV) identical to those computed on our real TMS-EEG data and described in the main text of this paper were then computed on this 'synthetic' data. Lastly, we compared statistically 'real' and 'synthetic' TMS-EEG data by applying whole-scalp cluster-based permutation (10000 permutations, $p<0.05$), similar to those included in the main manuscript, on values of inter-trial coherence (ITC) and imaginary Phase-Locking Value (PLV) in the high-beta band ([25 35] Hz), during a time-window corresponding to the TMS burst ([-133 0] ms).

This control analysis showed that high-beta ITC (Fig S3B) and right fronto-parietal imaginary PLV (Fig S3C) were significantly higher in magnitude during the stimulation window in 'real' *rhythmic* TMS-EEG datasets than in 'synthetic' data simulating a repetition of evoked single pulse TMS responses at a 30 Hz frequency. This outcome provides additional proof that the parietal

local ITC and inter-area phase synchronization increases during 30 Hz rhythmic TMS reported in our manuscript cannot be simply caused by a repetition of evoked-responses in close temporal succession.

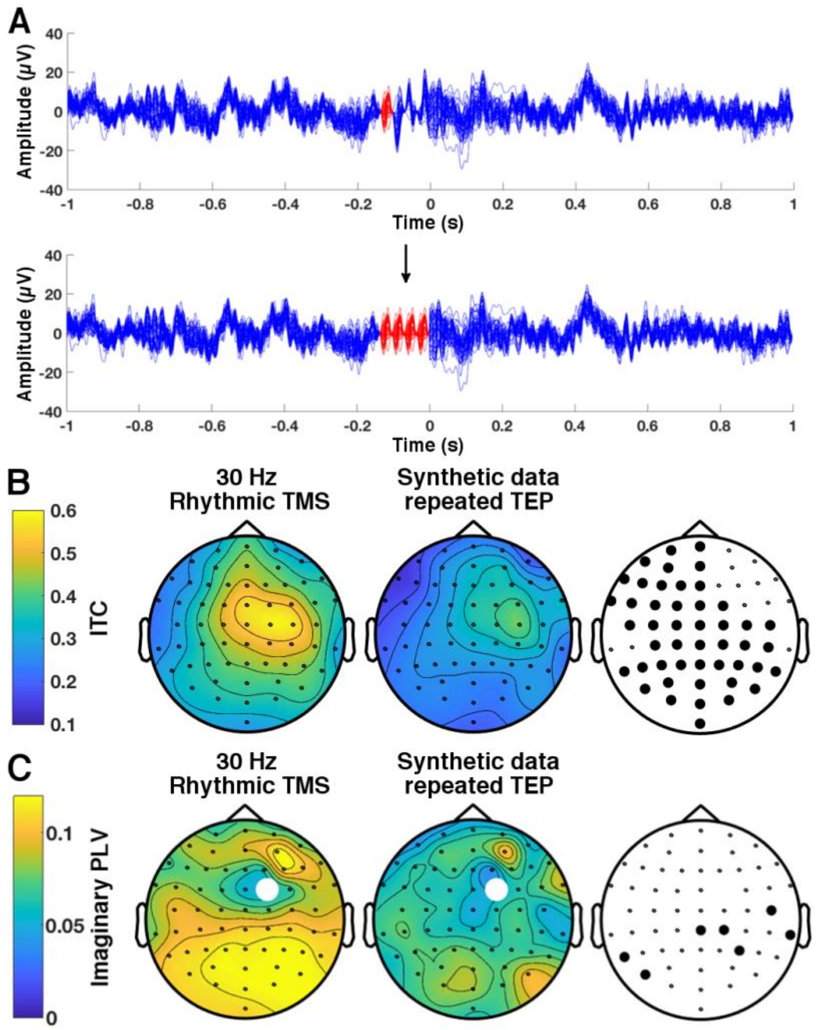

Figure S3: Control analysis to discriminate oscillatory entrainment from the impact of a series of periodically repeated TEPs. (A) We generated synthetic data simulating the repetition of Transcranial Evoked Potential (TEP) at 30 Hz. Epochs of EEG signals from each participant following the 1st TMS pulse in our *rhythmic* 4 pulse bursts were extracted (top graph, signal in red) from EEG traces and then repeated 4 times (bottom graph, signal in red). **(B)** Comparison of 30 Hz ITC during the TMS stimulation window ([-133 0] ms) between active rhythmic TMS-EEG data and the above-mentioned set of repeated single pulse 'synthetic' data. **(C)** Comparison of 30 Hz imPLV between electrode FC2 (labelled with a white dot, located closest to TMS stimulation site) and all other scalp electrodes during the TMS stimulation window ([-133 0] ms) between active rhythmic TMS-EEG data and repeated single pulse 'synthetic' data. **(B and C)** The statistical topographical map (right graphs) shows the outcomes of a pair-wise cluster-based permutation test for this comparison. Bolded electrodes represent clusters of contacts that reach statistical significance ($p<0.05$). This control analysis suggests that increases of high-beta ITC and imPLV during active rhythmic TMS cannot be solely caused by the repetition of TEPs at a 30 Hz frequency, but are likely related to a progressive trial-by-trial increase of oscillatory activity and oscillatory phase-locking at this frequency band throughout the stimulation burst.

Supplementary Results

Pre-TMS differences in oscillatory activity

In the left hemisphere, a statistical comparison between the active *rhythmic* and *random* TMS conditions revealed significant decreases of right frontal and left parietal imaginary PLV (measure of inter-area oscillatory synchrony) in the beta band emerging before TMS stimulation onset and prior to the presentation of a central alerting cue signaling the start of the next trial (time window [-0.3 -0.25] ms) (Fig. 3B in the main manuscript) (cluster T-stat = -87.774, p-value = 0.027, Cohen's d = 1.339).

Inter-trial coherence in the theta to alpha band was also significantly increased over left and right parietal contacts in the active *rhythmic* TMS condition compared to the active *random* TMS condition, prior to stimulation (time window [-0.3 -0.21] ms) (Fig. 5 in the main manuscript) (right hemisphere: cluster T-stat = 520.3, p-value = 0.0041, Cohen's d = 1.667; left hemisphere: cluster T-stat = 249.9, p-value = 0.0177, Cohen's d = 1.125).

Although unlikely, these significant differences could be associated to long-lasting effects of rhythmic and random stimulation, either alone or holding complex interactions with task events (e.g. visual target onset, motor response preparation and execution, or the onset of a central alerting cue indicating the start of a new trial and prompting subjects to fixate their gaze) hence inducing slightly different brain states throughout *rhythmic* and *random* stimulation blocks. Note however, that no significant pre-TMS time-frequency effects were observed for either imPLV or ITC when comparing active and sham trials randomly interleaved within the same experimental block.

Supplementary Figures

In order to better represent the data, we present additional figures which display EEG signal across time. First, we present average TEPs for all electrodes (butterfly plots in Fig S4, and their associated topographies during the stimulation period) or for selected right frontal (Fig S5A), right parietal (Fig S5B) and left parietal (Fig S5C) electrodes. Second, we present the waveform of evoked high-beta signal (EEG signal filtered in the [25 35] Hz band) across time, for selected right frontal (Fig S6A), right parietal (Fig S6B) and left parietal (Fig S6C) electrodes.

These figures show a progressive increase of evoked oscillatory high-beta activity across the 4 pulse TMS burst for active rhythmic stimulation, but not for random or sham stimulation, over right frontal and parietal regions.

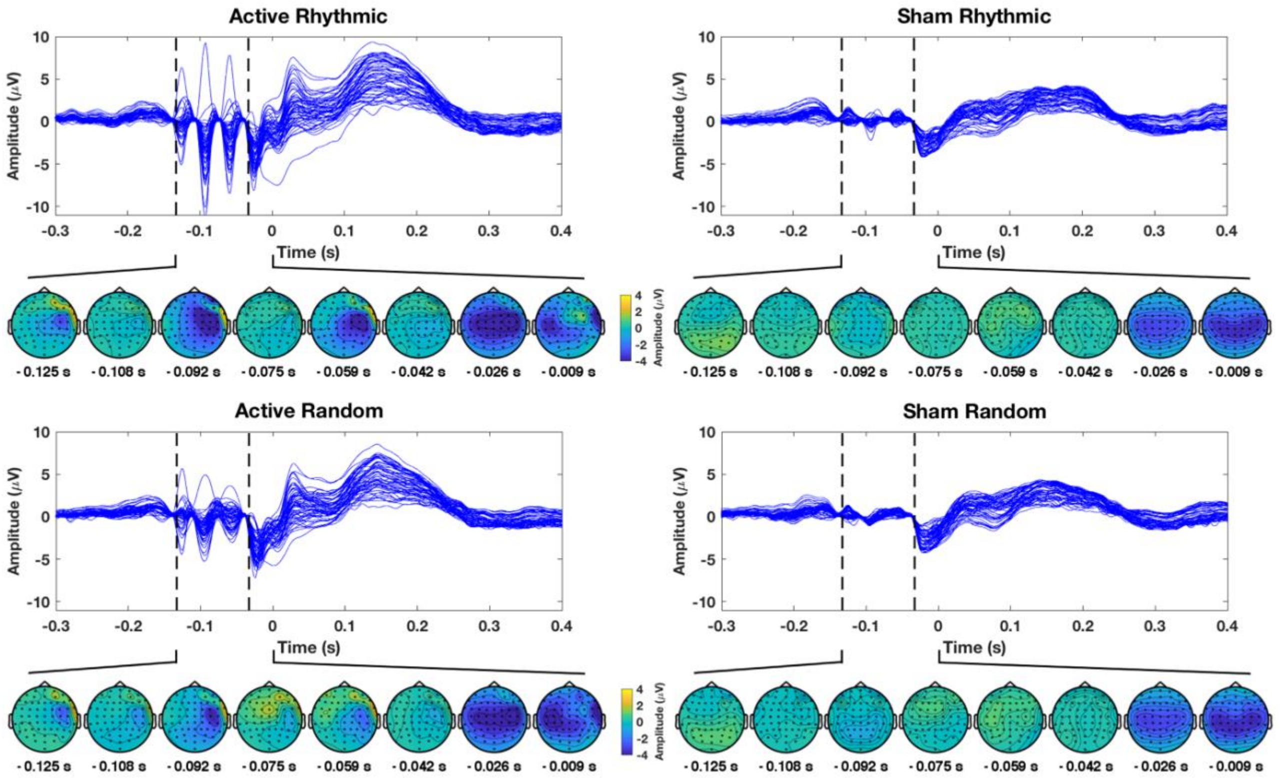

Figure S4. Butterfly plot of average TMS-evoked potentials and their topographies. Evoked potentials elicited by *active rhythmic*, *active random*, *sham rhythmic* and *sham random* stimulation. Black dotted vertical lines indicate the timing of the 1st and 4th TMS pulses during 4 pulse bursts. Time is centered (t=0) on the onset of the lateralized near-threshold visual target to be detected. Topographic data associated with evoked potentials were analyzed at 8 time-points during the duration of the TMS stimulation window [-0.133 0] s. Topographies were drawn for approximately the 90° and 270° phase of each of the four 30 Hz oscillation cycles encompassed by the TMS stimulation window.

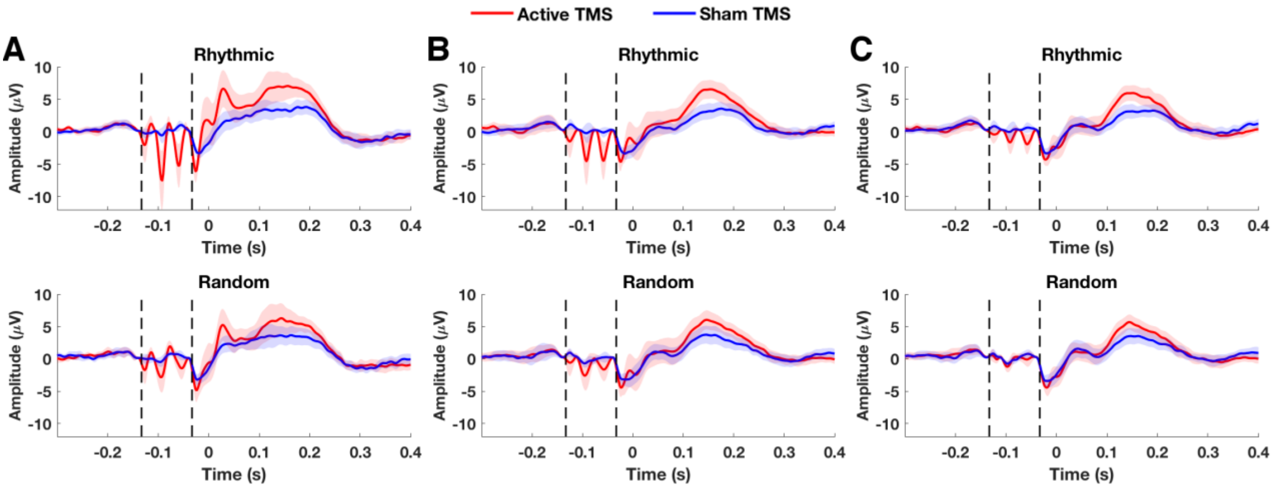

Figure S5. TMS-evoked potentials. Signals were baseline-corrected to a 700 ms time window preceding the apparition of the visual cue signaling the start of the trial and then averaged across trials and participants. Evoked potentials elicited by *rhythmic* (top graphs) and *random* (bottom graphs) active (red line) or sham (blue line) TMS patterns for: **(A)** electrode FC2 (closest to the center of the TMS coil); **(B)** A group of right parieto-occipital scalp electrodes; **(C)** a group of left parieto-occipital scalp contacts. Black dotted vertical lines indicate the timing of the 1st and 4th TMS pulses during 4 pulse bursts. Time is centered (t=0) on the onset of the lateralized near-threshold visual target to be detected. Colored shaded areas signal the 95% confidence interval for the amplitude of evoked potentials.

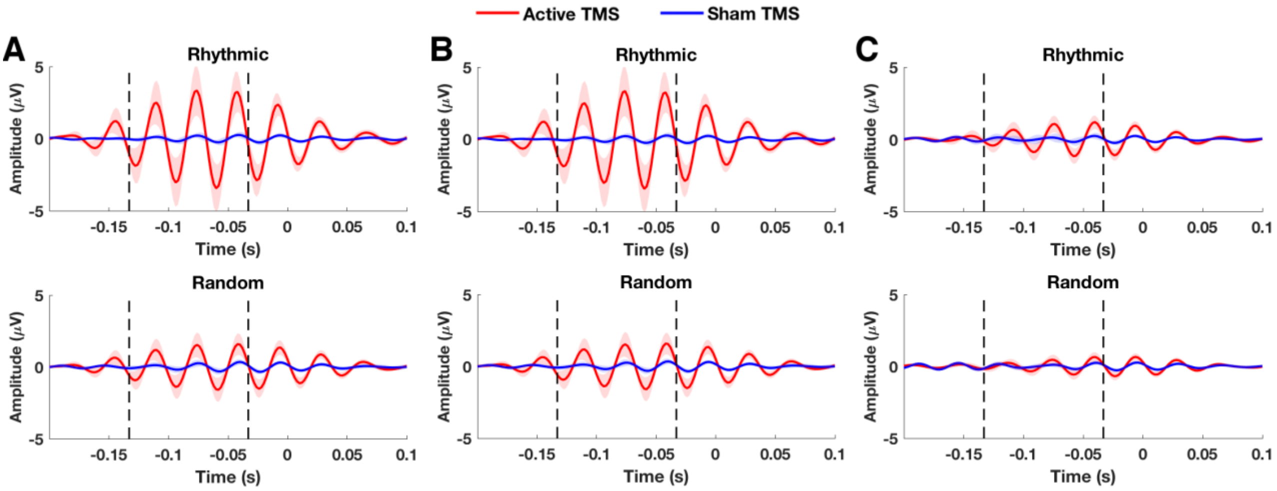

Figure S6. Average waveforms at 30 Hz. Signal were bandpass filtered [25 35] Hz before averaging across trials and participants. Waveforms associated to the *rhythmic* (top panels) and *random* (bottom panels) *active* (red lines) or *sham* (blue lines) TMS for: **(A)** Electrode FC2 (closest to the center of the TMS stimulation coil) ; **(B)** a group of right parieto-occipital scalp electrodes ; **(C)** A group of left parieto-occipital scalp contacts. Black dotted vertical lines indicate the timing of the 1st and 4th TMS pulses during 4 pulse bursts. Time is centered (t=0) on the onset of the lateralized near-threshold visual target to be detected. Colored shaded areas indicate 95% confidence interval for evoked oscillations amplitude.
